# Editorial: Enhancing T cell function: innovations in cancer immunotherapy

**DOI:** 10.3389/fimmu.2025.1615030

**Published:** 2025-05-06

**Authors:** Ji Zhang, Qihao Zhao, Qian Sun, Hong Jiang, Valentyn Oksenych, Xuefeng Wang

**Affiliations:** ^1^ Department of Ophthalmology, The Second Affiliated Hospital & School of Basic Medical Sciences, Soochow University, Suzhou, China; ^2^ Department of Clinical Laboratory, Yancheng Hospital of Traditional Chinese Medicine, Yancheng, China; ^3^ Cancer Institute and Hospital, Tianjin Medical University, Tianjin, China; ^4^ Department of Biotherapy, State Key Laboratory of Biotherapy and Cancer Center, West China Hospital & Department of Pancreatic Surgery, West China Hospital, Sichuan University, Chengdu, China; ^5^ Faculty of Medicine, University of Bergen, Bergen, Norway

**Keywords:** editorial: enhancing t cell function: innovations in cancer immunotherapy cancer immunotherapy, CD8 + T cells, stem-like CD8 + T cells, car-t, enhancing T cell function

Cancer immunotherapy has undergone great advancements over the past decade, with T cells emerging as central players in driving antitumor immunity. Despite remarkable clinical successes — particularly with immune checkpoint blockade (ICB) and adoptive T cell therapies like chimeric antigen receptor T (CAR-T) cells, T cell receptor T (TCR-T) cells, and tumor-infiltrating lymphocytes (TILs) — persistent challenges such as T cell exhaustion, limited persistence, and immunosuppressive tumor microenvironments (TME) continue to hinder therapeutic efficacy (1, 2).

This Research Topic compiles significant studies that address these barriers through effective strategies to enhance T cell function, offering new insights into molecular mechanisms, therapeutic engineering, and combinatorial approaches. We summarize key findings and their implications for the future of cancer immunotherapy as follows.

## Cancer immunotherapies can be enhanced through T-cell subpopulation insights

Recent studies deepen our understanding of T-cell heterogeneity across tumors. In chronic lymphocytic leukemia (CLL), mass cytometry of treatment-naïve patients identified three clinically relevant T-cell subpopulations: ICOS^+^HLA-DR^+^PD1^+^TIGIT^+^Tbet^+^CD4^+^ helper cells and two CD8^+^ subsets (CD27^+^CD28^-^PD1^+^Tbet^+^Eomes^+^; CD27^+^CD28^-^GrzmB^+^Tbet^+^Eomes^+^ terminal effectors). These subsets predicted reduced risks of secondary malignancies and mortality, validated across cohorts. Functional assays revealed enriched polyfunctional cytokine production in these subsets, positioning them as predictive biomarkers and therapeutic targets for optimizing chemoimmunotherapy outcomes (Lim et al.). Similarly, in diffuse large B-cell lymphoma (DLBCL), CD73^+^CD8^+^ T cells exhibited enhanced cytotoxicity with reduced exhaustion markers, suggesting their role as reservoirs of functional antitumor immunity (Zhang et al.). These findings underscore the importance of dissecting T-cell heterogeneity to refine patient stratification and therapy design.

## Chemotherapeutic agents potentiate long-term antitumor immunity by expanding stem-like CD8^+^ T cell populations

**Figure 1:**
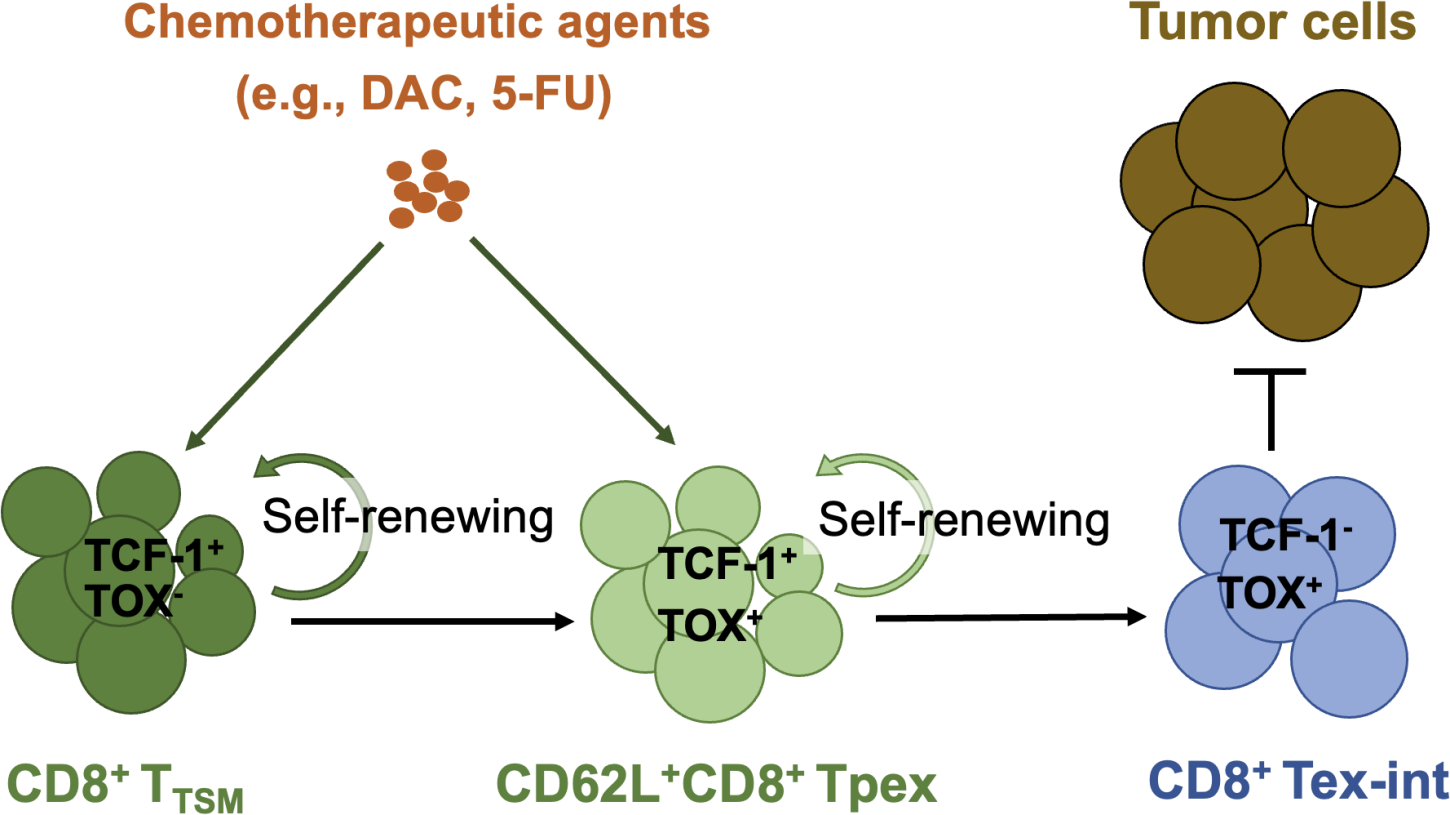
The distinct roles of chemotherapeutic agents (e.g., DAC, 5-FU) in enhancing antitumor immunity via expanding stem-like CD8^+^ T cell subsets. T_TSM_, Tumor-specific memory CD8^+^ T cells; Tpex, Precursors of exhausted CD8^+^ T cells; Tex-int, Transitory effector-like exhausted CD8^+^ T cells; DAC, Decitabine; 5-FU, 5-Fluorouracil.

Stem-like CD8^+^ T cells play a pivotal role in sustaining durable immune responses (3). These cells, characterized by self-renewal capacity and the ability to differentiate into effector subsets, are critical for overcoming T cell exhaustion. Ruan et al. demonstrated that chemotherapeutic agents such as decitabine (DAC) and 5-fluorouracil (5-FU) expanded tumor-specific memory CD8^+^ T (T_TSM_) cells in tumor-draining lymph nodes and enhanced the differentiation of CD62L-expressing exhausted precursor CD8^+^ T (Tpex) cells into functional CX3CR1-expressing effector-like intermediate exhausted T (Tex-int) cells within tumors**^1^** (**Figure 1**). Notably, DAC’s effects were partially dependent on the transcription factor Eomes, highlighting a targetable pathway for amplifying stem-like T cell populations (Ruan et al.). These findings align with broader efforts to harness stem-like T cells, as seen in strategies combining epigenetic modulation or metabolic reprogramming with ICB (4).

Moreover, Liu et al. reviewed the discovery of RUNX family transcription factors as regulators of CD8^+^ T cell infiltration in colorectal cancer underscores the potential of targeting transcriptional networks to enhance T cell longevity and function. Such approaches could synergize with therapies aimed at preserving stem-like phenotypes, offering a dual mechanism to counteract exhaustion (Liu et al.).

## Engineering next-generation T cell therapies are revolutionizing cancer immunotherapy

Adoptive cell therapies (ACT) have revolutionized oncology, yet limitations in persistence, scalability, and safety remain. This Research Topic showcases cutting-edge engineering solutions as follows.

First, Zhang et al. reported a universal CAR-T (UCAR-T) with self-activated protection. An anti-CD70 UCAR-T platform incorporating a “self-activated and protective” (SAP) module — comprising CD47 for immune evasion and IL-7Rα for survival — demonstrated enhanced persistence and reduced host rejection in preclinical models. This innovation addresses key hurdles in allogeneic therapies, paving the way for scalable “off-the-shelf” products (Zhang et al.).

Next, Wang et al. proved that integration of thermotherapy and Traditional Chinese Medicine (TCM) synergistically enhanced antitumor immune responses. Heating Jurkat T cells to 39°C augmented their cytotoxicity against tumors by inducing heat shock proteins and mitochondrial activation. Coupled with TCM components like Shengmai Injection, this approach amplified T cell proliferation and downregulated apoptotic pathways. Such combinatorial strategies highlight the untapped potential of integrating biophysical stimuli with pharmacologic agents (Wang et al.).

Then, Ye et al. substantiated that a kind of fusion protein for targeted IL-2 delivery, AWT020 fusion protein, which combines a PD-1-blocking nanobody with an engineered IL-2 mutein, selectively expanded tumor-infiltrating CD8^+^ T cells while minimizing systemic toxicity**^3^**. This precision delivery system exemplifies how modular designs can enhance therapeutic indices (Ye et al.).

These advances are complemented by broader trends in gene editing (e.g., CRISPR-based enhancements) and nanomedicine (5), which enable precise modulation of T cell behavior and microenvironmental interactions.

## Immunomodulatory reprogramming of the tumor microenvironment potentiates antitumor immunity

The TME’s immunosuppressive nature remains a formidable barrier. Studies here emphasize dual strategies: disrupting inhibitory signals and reprogramming stromal components.

Targeting metabolic and mechanical cues. Dysregulated nucleotide metabolism in lung adenocarcinoma (LUAD) was linked to poor prognosis and immune evasion, with the oncogene AUNIP promoting tumor progression and suppressing CD8^+^ T cell infiltration. Similarly, tissue stiffness in solid tumors was shown to impede immune cell trafficking, underscoring the need for metabolic modulatory therapies (Luo et al.).

Repurposing natural immunomodulators. β-glucan, when combined with camrelizumab (anti-PD-1) and chemotherapy, enhanced IL-2 and IFN-γ levels in gastric cancer patients, correlating with prolonged survival (Chu et al.). This aligns with efforts to leverage innate immune activators as adjuvants for ICB.

Pericytes and immune Crosstalk. In esophageal squamous cell carcinoma (ESCC), heterogeneous cancer-associated pericytes (CAPs) were found to interact dynamically with immune cells, with specific subtypes serving as prognostic biomarkers and therapeutic targets (Xiong et al.). Such insights reveal the TME’s complexity and the need for multiplexed targeting.

## Biomarkers drive personalized antitumor immunotherapy

The integration of multi-omics and single-cell technologies has accelerated biomarker discovery. For example, high RUNX1/3 expression in CD8^+^ and CD103^+^CD8^+^ T cells in colorectal cancer correlated with improved outcomes, suggesting their utility as predictive biomarkers for immunotherapy response (Liu et al.). In gastric cancer, NALCN loss impaired T cell proliferation and altered metabolite uptake, positioning it as both a prognostic marker and a modulator of immune cell recruitment (Li et al.).

These studies highlight the shift toward precision oncology, where patient stratification based on immune and molecular profiles will optimize therapeutic selection.

In summary, the innovations highlighted in this Research Topic collectively advance our understanding of T cell biology and its therapeutic manipulation. From engineering resilient CAR-T cells to reprogramming the TME, these studies exemplify the multidisciplinary effort required to overcome immunotherapy’s current limitations. As the field moves toward personalized, biomarker-driven regimens, the integration of synthetic biology, nanotechnology, and computational modeling will be indispensable. By bridging basic science and clinical translation, we inch closer to a future where durable remissions in solid and hematologic malignancies become the norm rather than the exception.
